# Re-Irradiation for Locally Recurrent Lung Cancer: A Single Center Retrospective Analysis

**DOI:** 10.3390/curroncol28030170

**Published:** 2021-05-13

**Authors:** Brane Grambozov, Evelyn Nussdorfer, Julia Kaiser, Sabine Gerum, Gerd Fastner, Markus Stana, Christoph Gaisberger, Romana Wass, Michael Studnicka, Felix Sedlmayer, Franz Zehentmayr

**Affiliations:** 1 Department of Radiation Oncology, Paracelsus Medical University, SALK, A-5020 Salzburg, Austria; evelyn.nussdorfer@stud.pmu.ac.at (E.N.); j.kaiser@salk.at (J.K.); s.gerum@salk.at (S.G.); g.fastner@salk.at (G.F.); m.stana@salk.at (M.S.); c.gaisberger@salk.at (C.G.); f.sedlmayer@salk.at (F.S.); f.zehentmayr@salk.at (F.Z.); 2 Department of Pneumology, Paracelsus Medical University, SALK, A-5020 Salzburg, Austria; r.wass@salk.at (R.W.); M.Studnicka@salk.at (M.S.); 3 Department of Pulmonology, Kepler University Hospital, A-4020 Linz, Austria; 4 radART—Institute for Research and Development on Advanced Radiation Technologies, Paracelsus Medical University, A-5020 Salzburg, Austria

**Keywords:** lung cancer, local recurrence, reirradiation, PTV, overall survival

## Abstract

The treatment of locally recurrent lung cancer is a major challenge for radiation-oncologists, especially with data on high-dose reirradiation being limited to small retrospective studies. The aim of the present study is to assess overall survival (OS) for patients with locally recurrent lung cancer after high-dose thoracic reirradiation. Thirty-nine patients who were re-irradiated for lung cancer relapse between October 2013 and February 2019 were eligible for the current retrospective analysis. All patients were re-irradiated with curative intent for in-field tumor recurrence. The diagnostic work-up included a mandatory ^18^F-FDG-PET-CT scan and—if possible—histological verification. The ECOG was ≤2, and the interval between initial and second radiation was at least nine months. Thirty patients (77%) had non-small cell lung cancer (NSCLC), eight (20%) had small cell lung cancer (SCLC), and in one patient (3%) histological confirmation could not be obtained. More than half of the patients (20/39, 51%) received re-treatment with dose differentiated accelerated re-irradiation (DART) at a median interval of 20.5 months (range: 6–145.3 months) after the initial radiation course. A cumulative EQD_2_ of 131 Gy (range: 77–211 Gy) in a median PTV of 46 mL (range: 4–541 mL) was delivered. Patients with SCLC had a 3 mL larger median re-irradiation volume (48 mL, range: 9–541) compared to NSCLC patients (45 mL, range: 4–239). The median cumulative EQD2 delivered in SCLC patients was 84 Gy (range: 77–193 Gy), while NSCLC patients received a median cumulative EQD2 of 135 Gy (range: 98–211 Gy). The median OS was 18.4 months (range: 0.6–64 months), with tumor volume being the only predictor (*p* < 0.000; HR 1.007; 95%-CI: 1.003–1.012). In terms of toxicity, 17.9% acute and 2.6% late side effects were observed, with a toxicity grade >3 occurring in only one patient. Thoracic high dose reirradiation plays a significant role in prolonging survival, especially in patients with small tumor volume at recurrence.

## 1. Introduction

Lung cancer is one of the most common and deadly malignancies worldwide, with 276.000 estimated deaths in the European Union alone in 2019 [1]. Up to 40% of lung cancer patients, who undergo chemo-radiation, experience local recurrence within two years after treatment [2,3,4,5,6,7,8], which may partly be due to their improved life expectancy. Historically, re-irradiation of lung cancer patients began in the early 1980s with the study by Green and Melbye [9]. In a cohort of 29 patients, most of whom received total doses between 20 and 40 Gy, the authors demonstrated a survival of five months after the second radiation course. In 2003, a small prospective study of 23 patients showed a median OS (mOS) of 15 months after re-treatment [5]. In the following years, the development of intensity modulated radiotherapy (IMRT), volumetric intensity modulated arc therapy (VMAT), and stereotactic ablative body radiotherapy (SABR) facilitated dose escalation to the tumor with less fear of toxicity. Hence, the mOS for patients with recurrent lung cancer rose to 20 months and beyond after radical re-irradiation [6]. Retrospective studies including at least one course of SABR demonstrated higher mOS compared to those without stereotactic techniques, implying that patients with low recurrent disease burden, in particular, could significantly benefit from locally ablative treatment approaches [6].

Despite these advances, treating recurrent lung cancer patients is still a major challenge, especially given the lack of data on toxicity. Side effects such as bleeding, radiation pneumonitis, esophagitis, and radiation-induced myelopathy remain of concern, particularly as these patients are prone to treatment complications because of pre-existing co-morbidity. While some series reveal a considerable rate of clinically relevant side effects (i.e., grade 3 and 4) of approximately 20% and up to 12% lethal bleedings [10], others report no adverse event higher than grade 2 [5,11,12,13] (reviewed by De Ruysscher [6]). Since chemotherapy has limited success with mOS rates in the range of one year at best [6], re-irradiation could play a more important role in the treatment of patients with recurrent lung cancer.

The aim of this single center retrospective analysis is to evaluate OS after thoracic re-irradiation for patients with locally recurrent lung cancer and to identify factors that might influence clinical outcome.

## 2. Methods

### 2.1. Patients

Between October 2013, which marks the implementation of IMRT/VMAT for high-dose thoracic irradiation at our institute, and February 2019, 39 patients, who met the following inclusion criteria, underwent thoracic re-irradiation: (1) The primary as well as the secondary tumor were located in the lung. (2) All patients were classified as inoperable and received two courses of curatively intended radiation therapy. (3) If possible, the tumor was histologically verified and categorized according to the 8th edition of the TNM classification. (4) ^18^F-FDG-PET-CT was required in the diagnostic work-up. (5) The performance status had to be ≤2 according to the Eastern Cooperative Oncology Group (ECOG). (6) The time interval between initial and secondary radiation therapy was nine months or more. Patients receiving palliative treatment, postoperative radiotherapy (RT), or those with chest wall tumors and / or out-of-field recurrences were excluded. Each case was discussed in a multidisciplinary tumor board with pneumologists, medical oncologists, radiologists, thoracic surgeons, pathologists, and radiation oncologists.

### 2.2. Radiation Therapy

Prior to IMRT/VMAT, a planning CT scan with an acquisition time of three seconds was performed. Additionally, 4D-CT was performed in SABR patients. A vacuum cradle and WingSTEP^TM^ were used for immobilization. Subsequently, the planning CT was co-registered with the ^18^F-FDG-PET-CT. For SABR patients, the ITV was created by contouring the GTV on three breathing phases (expiration, inspiration, and average) and their subsequent union (ITV = CTV). The PTV was generated by adding a symmetric margin of 5 mm to the ITV. For IMRT/VMAT patients, the GTV was contoured on a so-called “slow CT” with an acquisition time of three seconds. This GTV represents an ITV/CTV because it includes the respiration-dependent movement of the tumor. The planning target volume (PTV) was defined by adding a margin of 7 mm symmetrically to GTV. IMRT/VMAT was delivered in three fractionation regimens: dose-differentiated accelerated RT in twice daily fractions of 1.8 Gy (DART-bid), as described in two previous publications [14,15], conventionally with 2 Gy per fraction, and hypofractionated RT (one fraction of 3 Gy per day). SABR was delivered in two different schemes: 8 fractions of 8 Gy (65% isodose) delivered daily for central tumors (i.e., within 2 cm of the proximal bronchial tree) and 3 fractions of 15.4 Gy on (65% isodose) every other day for peripheral tumors. Organs at risk (OAR), such as the esophagus, central vessels and airways, spinal cord, lungs, and heart were routinely contoured, and dose volume histograms of both initial and re-irradiation plans were used to determine the cumulative radiation exposure of each critical organ. On the basis of a previous evaluation of long-term toxicity after a single course of high-dose chemo-radiation, which revealed that a dose maximum of 80 Gy to the esophagus was associated with a 33.5% clinically relevant grade 2 or 3 toxicity, the D_max_ was set at 100 Gy [12,16]. Okamoto et al. showed in a small cohort of long-term survivors that a D_max_ of 110 Gy to trachea and main bronchi can be well tolerated, and therefore we adopted the same constraints [17]. For the spinal cord, a D_max_ of 75 Gy was applied [18]. The maximum volume that could receive more than the above-specified cumulative dose was set at 1 mL. As for the lungs, a V20_total lung_ < 50% was used on the basis of a previous publication by our group [16]. Regarding cardiac toxicity, QUANTEC suggests dose constraint of V25_heart_ < 10%, which is associated with a risk of 1% cardiac mortality in 15 years [19]. Given the lack of alternative treatment options and limited prognosis for patients with loco-regionally recurrent lung cancer, the constraint was set at twice this value, i.e., a V25_heart_ of 20%.

### 2.3. Biologically Equivalent Dose

Since various fractionation regimens were used, total radiation doses were compared by a biologically equivalent dose in 2 Gy fractions (EQD_2_). Under the assumption of an *α/β =* 10 for the tumor, the following algorithm was used:

$$EQD2=D\;\;x\;\frac{d+\alpha /\beta }{2+\alpha /\beta }$$

*D* is the total radiation dose including both treatment courses and *d* is the single dose. Since the overall treatment time (OTT) of the second radiation course was less than the reference time for the start of accelerated repopulation (T_ref_ = 28 [20]) in most cases, the time factor would have been zero and was therefore not included in the calculation. Additionally, it remains unclear how to calculate a cumulative EQD_2_ with at least nine months interval between treatment courses.

### 2.4. Systemic Treatment

Prior to re-irradiation, patients received a platinum-doublet if they were medically fit and fulfilled at least one of the following criteria: lesion size ≥ 4 cm, centrally located tumor or positive mediastinal or hilar lymph nodes. The regimen for non-small cell lung cancer (NSCLC) consisted of two cycles of either cisplatin (75 mg/m2/d) combined with pemetrexed (500 mg/m2/d) or gemcitabine (1000 mg/m2/d), while small-cell lung cancer (SCLC) patients received four cycles of one of the aforementioned platinums together with etoposide (120 mg/m2 days 1–3). In case of renal dysfunction dysfunction, carboplatin at an area under the curve (AUC) of 5 on day 1 (absolute maximum dose 1100 mg) was applied instead of cisplatin. Of the 39 patients, 7 (18%) received chemotherapy only, 14 (36%) received immunotherapy only, and 3 (7.7%) patients received both chemotherapy and immunotherapy. Depending on the tumor histology, patients received one of the following immunotherapeutic agents after the re-irradiation: atezolizumab, nivolumab durvalumab, or pembrolizumab.

### 2.5. Follow Up

The patients were followed up six weeks after the end of re-irradiation, then every three months for the first two years and twice a year thereafter. The follow-up visits included clinical examination, contrast-enhanced CT, and pulmonary function test. On suspicion of local recurrence or new lung lesions in the chest CT, an ^18^F-FDG-PET-CT was performed. Missing data were retrieved from the Austrian death registry and general practitioners.

### 2.6. Toxicity

Toxicity was reported according to the Common Terminology Criteria for Adverse Events version (CTCAE) 4.03, focusing on esophagitis, pneumonitis, and bleeding as the most important side effects caused by thoracic irradiation. Grade 1 toxicities were not assessed, as they were not considered clinically relevant. For differentiation between acute and late toxicities, a cutoff of 90 days as of completion of re-irradiation was used, except for pneumonitis, which was regarded still as acute if it occurred within 180 days after the end of RT.

### 2.7. Statistics

The primary endpoint of the current study was OS after thoracic re-irradiation. Clinical outcome (OS and LC) was calculated with the Kaplan–Meier method. OS was defined as the time between the end of re-irradiation and death or latest follow-up. Local relapse was defined as tumor growth within the re-irradiated volume covered by the 95%- or 65%-isodose after IMRT/VMAT or SABR, respectively. Subgroups were compared by the log-rank test. In order to identify parameters that potentially influence OS, a multivariate model (MVA, Cox regression, forward stepwise) with the following patient characteristics and treatment related factors was calculated: age, sex, weight loss, ECOG, histology, T-stage, N-stage, M-stage, FEV1, COPD grade, Charlson co-morbidity index (CCI), re-irradiation volume, tumor location, cumulative EQD_2_, interval between radiation courses, V20_total lung_, V25_heart_, local control, and systemic treatment.

## 3. Results

### 3.1. Patients

The total cohort included 25/39 (64%) men and 14/39 (36%) women. The median age at the start of re-irradiation was 66 years (range: 52–83 years). According to histological findings at initial diagnosis, 30/39 (77%) patients had NSCLC, and 8/39 (20%) patients had SCLC. In one patient (3%), no pathological confirmation could be obtained. The patient with unknown histology is listed as such in Table 1 and was included in the calculation made for NSCLC patients. The majority of patients with NSCLC (27/31, 87%) and SCLC (6/8, 75%) had an ECOG performance score ≤ 1. The mean CCI was 6 (NSCL 6; SCLC 8). Most of the patients (22/39, 56%; NSCLC 17/31, 55%; SCLC 5/8, 63%) had stage III disease. Seven patients (18%), five diagnosed with NSCLC and two with SCLC, had oligometastatic UICC IV on re-irradiation. Two of these patients already presented with a single metastasis at initial diagnosis: one had a nodule in the contralateral lung and the other in the right adrenal gland. Five patients who initially presented with stage III disease progressed to oligo-metastatic UICC stage IV in the interval between initial treatment and re-irradiation (Table 1).

### 3.2. Re-Irradiation

In 22/39 (56%; NSCLC 15/31, 48% versus SCLC 7/8, 88%) of the patients, the tumor was centrally located, whereas in the other 17/39 (44%; NSCLC 16/31, 52% versus SCLC 2/8, 23 %) cases, it was peripheral. As for the re-irradiation technique and fractionation, DART-bid was applied in 20/39 patients (NSCLC 17/39, SCLC 3/39), while 10/39 (NSCLC 9/39, SCLC 1/39) received SABR. Five patients (NSCLC 2/39, SCLC 3/39) received hypofractionated RT and 4/39 (NSCLC 2/39, SCLC 2/39) conventional RT. The median time interval between the first and second radiation treatment was 20.5 months (range: 6–145.3 months). The median time until re-irradiation was 16 months (range: 6–145) in patients with SCLC, while it was 21 months (range: 9–80) in patients with NSCLC. In the whole cohort, the median re-irradiation planning target volume (PTV) was 46 mL (range: 4–541 mL), and the median cumulative EQD_2_ in both treatment courses was 131 Gy (range: 77–211 Gy). Patients with SCLC had a 3 mL larger median re-irradiation volume (48 mL, range: 9–541) compared to NSCLC patients (45 mL, range: 4–239). The median cumulative EQD2 delivered in SCLC patients was 84 Gy (range: 77–193 Gy), while NSCLC patients received a median cumulative EQD2 of 135 Gy (range: 98–211 Gy). In ten patients, at least one course of SABR was applied, resulting in a median EQD_2_ of 149 Gy (range: 135–211 Gy). In the non-SABR group, the median EQD_2_ was 119 Gy (range: 77–150 Gy). Eighteen patients (46%) had two cycles of a platinum-doublet prior to re-irradiation (Table 1). Information on dose, fractionation, and systemic therapy at first irradiation are listed in Table 1.

### 3.3. Overall Survival and Local Control

**Figure 1:**
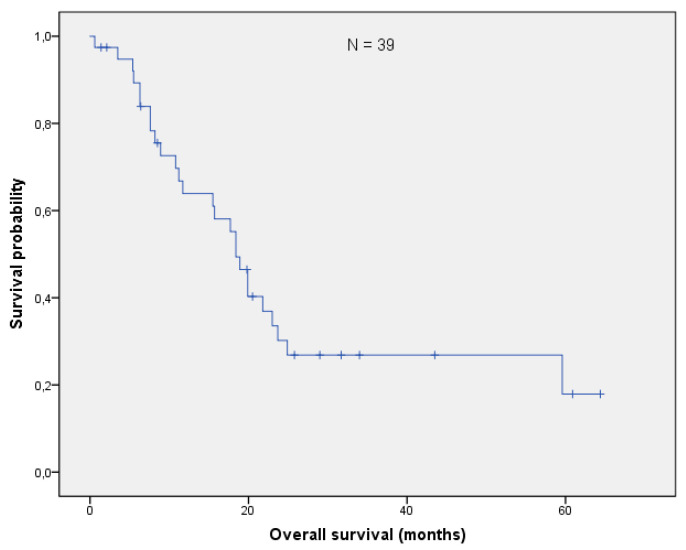
The median overall survival in the whole cohort (N = 39) was 18.4 months (95% CI: 16.0–20.8).

**Figure 2:**
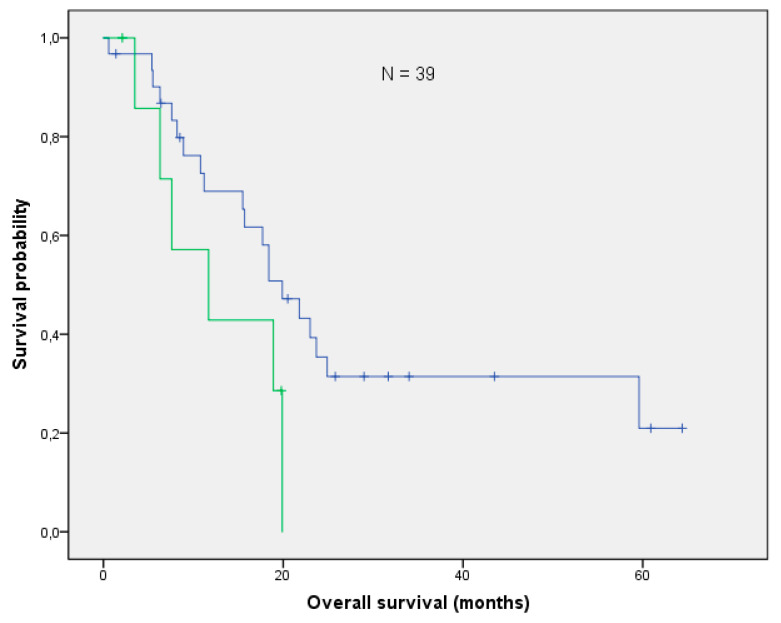
The median overall survival between patients with NSCLC (N = 31) and SCLC (N = 8) did not differ significantly (log-rank *p*-value: *p* = 0.101).

**Figure 3:**
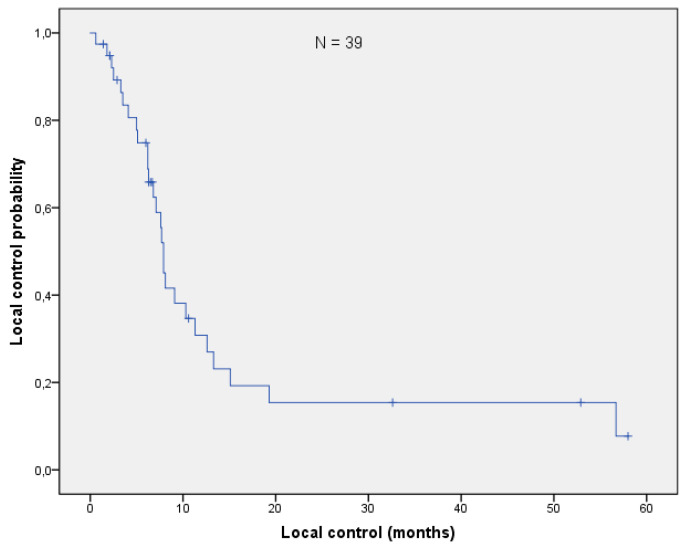
The median local control was 7.9 months (95% CI: 7.3–8.5).

**Figure 4:**
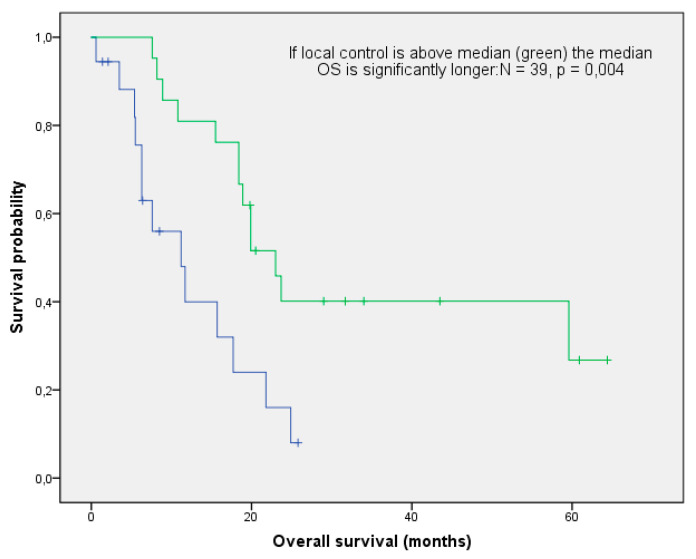
Impact of local control on overall survival: patients with local control above median (green) had a significantly longer OS (log-rank *p*-value: 0.004).

With a median follow-up of 17.7 months (range: 0.6–64.4 months), the mOS after re-irradiation was 18.4 months (range: 0.6–64.4 months; Figure 1). Thirteen patients (33%) are still alive, while 22/39 (54%) had cancer-related deaths. Three patients died from one of the following reasons: cardiac failure or exacerbation of COPD and peritonitis. In one patient, re-irradiation could have potentially been the cause of death (see toxicity). With respect to histology, no difference in OS could be found (log-rank *p*-value = 0,101; Figure 2). The median local control was 7.9 months (range 0.6–58 months; Figure 3). If long-term local control could be achieved, the patients had a significant benefit in terms of OS (log-rank *p*-value = 0.004; Figure 4). In the univariate analysis (UVA), LC (*p* = 0.005) was also one of the three parameters that were found to potentially influence OS—together with M-stage (*p* = 0.015) and re-irradiation volume (*p* < 0.000). However, in MVA (Cox regression, forward stepwise) the volume at re-irradiation (median: 46 mL, range: 4–541 mL) remained the only significant variable (*p* < 0.000; HR 1.007; 95%-CI: 1.003–1.012). A full list of model parameters and the respective *p*-values are shown in Table S1.

### 3.4. Toxicity

In general, thoracic re-irradiation was well tolerated. Acute toxicity grade 2 and 3 was observed in 5/39 (13%) and 2/39 (5%) patients, respectively. One patient died of cardio-respiratory failure one week after the completion of re-irradiation without any history of cardiac morbidity. In this patient, the cumulative maximum EQD_2_ in both radiation courses was 110 Gy, which was below the 115 Gy reported in literature as tolerable D_max_ [12]. The V20_total lung_ of 43% met the above-mentioned constraint, while the V25_heart_ of 45% did not because the tumor was located in the central upper lobe including the left hilum and upper segments of the lower lobe. Hence, this patient was evaluated as having acute grade 5 cardiac toxicity. Only one patient (1/39, %) experienced late side effects in terms of grade 3 hemorrhage, which had no further consequences after blood transfusion (see Table 2). The cumulative doses to organs at risk (OAR) are summarized at the end of Table 1 and Table S2.

## 4. Discussion

The current analysis demonstrates that re-irradiation in patients with good PS yields one-month mOS, with small irradiation volume as the most important predictive factor. Long-term LC was also found to positively influence survival after re-irradiation.

According to the literature, the expected mOS after curatively intended re-irradiation is 17.7 months (range: 7.1 [21] to 31.4 [4] months) [6,7]. Hence, it seems that in a small portion of selected patients even long-term survival can be achieved. It is noteworthy that mOS rates of 20 months and beyond are reached in small recurrent tumors that can be treated with SABR [6]. In the current cohort, however, the majority of patients presented with locally advanced NSCLC, which entails higher tumor burden, larger PTVs and more doses to the OARs compared to SABR. Apart from the exceptionally high mOS in the study by Sumita [4], the current cohort seems to be on the upper edge of the achievable outcome range. In this closely monitored cohort with contrast enhanced chest CT scans every three months, the median LC after re-irradiation was 7.9 months, which was in the same range as in the study by Schlampp [22] but rather poor compared to the previously mentioned Japanese study, which reported 12.9 months loco-regional control [4]. This can be partly attributed to the fact that in the study by Sumita [4], the loco-regional control was calculated from the first day of re-irradiation to the earliest point of local failure, in contrast to our study in which the loco-regional control was calculated from the end of re-irradiation. While LC turned out to be a predictive parameter for OS in univariate analysis, PTV was highly significant on MVA (*p* < 0.000; HR 1.007; 95%-CI: 1.003–1.012). Three other retrospective studies corroborate this outcome by reporting a significant correlation between PTV and OS [4,10,23]. As a small radiation volume seems to be a predictive factor for good OS after re-irradiation, a close follow-up after initial treatment is highly reasonable. In our cohort, this practice resulted in a median PTV of 46 mL (NSCLC 45 mL, SCLC 48 mL), which is about one-fifth the average size of the previously mentioned three reports [4,10,23]. This is in line with the radiobiological notion that ionizing radiation is more effective in small tumor nodules because of better oxygenation and lower mutational burden [24]. Moreover, in such cases a higher EQD_2_ can be delivered, while sparing OARs, as already published in previous series on SABR [23,25,26,27,28,29]. A potential correlation between a higher radiation dose and better OS, portrayed in studies with palliative, conventional, and stereotactic re-irradiation by the significant difference in mOS, has already been discussed in two reviews [6,8]. When excluding the ten patients in our analysis who received at least one course of SABR, the median cumulative EQD_2_ is still approximately 20% higher than in the cohort published by Schlampp [22], which might explain the comparatively high mOS in our patients.

With retrospective analyses like this, one may argue that the inherent selection bias tampers with clinical outcome. In fact, the current cohort was comparable to other studies in terms of performance score (PS) and time interval between the two radiation courses [4,5,10,17,22,23]. Reports on radical re-irradiation usually include patients with PS ≤2 because it directly affects OS [21,30]. Higher PS implies difficulties to complete RT, which negatively influences clinical outcome [4,5,10,17,21,22,23,31,32]. Additionally, two retrospective analyses showed that a longer time span between radiation treatments plays a significant role in improving OS [21,33]. According to the review by Rulach [7], the median interval in most studies is about 23 months, which is two months longer than in the present study. Although in MVA neither PS nor the interval between radiation courses reached significance, it is not far-fetched to assume that these factors exert influence on outcome by regulating total dose to OARs.

Of note, the rather high median cumulative dose in the current analysis is associated with comparatively low complication rates. We observed clinically relevant acute side effects (i.e., esophagitis and pneumonitis) grade 2 and 3 of 12.8% and 5.1%, respectively (Table 2). Esophagitis was predominant probably because of the central tumor location in almost 60% of the patients, which corroborates findings in a previously published set of 38 patients [3]. One patient (2.6%) had a grade 3 hemorrhage, which was the only late adverse event (Table 2). A possible explanation for the favorable toxicity profile despite the high cumulative EQD_2_ might be the tight margins added to the GTV. Similar to previously published analyses on thoracic re-irradiation using techniques other than SABR [3,22], one case of potential grade 5 toxicity was observed in the current cohort. As summarized in a review by De Ruysscher, lethal side-effects mainly occur in patients with centrally located tumors [6].

The current analysis is limited by its retrospective nature. As the value of thoracic re-irradiation in terms of clinical outcome is yet to be determined, cohorts like this add to the database of potential treatment options, especially since the current report mainly included patients with centrally located tumor recurrences. The small number of patients–similar to other studies in the field—is also the reason why the two major histology types (NSCLC/SCLC) of lung tumors were included in the very same analysis. Nevertheless, the inclusion of both non-small cell and small cell lung cancer was not statistically relevant in terms of OS (Figure 2, Table S1). For lack of prospective studies on thoracic re-irradiation, our data may contribute to clinical experience in spite of these above-mentioned shortcomings.

## 5. Conclusions

Thoracic high dose re-irradiation plays a significant role in prolonging survival particularly in patients with small tumor volumes, thus warranting close follow up periods after initial treatment. Prospective studies on radical re-irradiation with standardized data collection are needed.

## Figures and Tables

**Table 1:** Patients and treatment. Abbreviations: EQD_2_: biologically equivalent dose in 2 Gy fractions, V20_total lung_: total lung volume that receives 20 Gy or more, V25_heart_: heart volume that receives 25 Gy or more; * including one patient with unknown histology.

Patient Characteristics and Treatment Factors			NSCLC N = 31	SCLC N = 8
Patient Characteristics	Age (years)	median	66.5	66.1
		range	52–83	52–73
	Sex	male	20	5
		female	11	3
	Weight loss (%)	>5%	17	5
		<5%	14	3
	ECOG	0–1	27	6
		2	4	2
	Histology	SCLC	0	8
		NSCLC	30	0
		unknown	1 *	0
	T-stage	x	2	1
		1	6	1
		2	16	0
		3	4	4
		4	3	2
	N-stage	0	7	0
		1	4	2
		2	16	4
		3	4	2
	M-stage	0	26	6
		1	5	2
	UICC-stage	I	4	0
		II	5	1
		III	17	5
		IV	5	2
	FEV1 (%)	median	72	70
		range	29–100	35–100
	COPD grade	0	13	1
		1	2	0
		2	2	6
		3	9	0
		4	4	1
		unknown	1	0
	Charlson Comorbidity Index	median	6	9
		range	3–10	4–10
Treatment Related Factors	Re-rad volume (mL)	median	45	48
		range	4–239	9–541
	Tumor location at re-rad (*n*)	peripheral	16	1
		central	15	7
	EQD2 (Gy) at first radiation	median	77	47
		range	50–88	40–60
	EQD2 (Gy) at re-rad	median	61	46
		range	44–133	40–50
	Cumulative EQD_2_ (Gy)	median	135	84
		range	98–211	77–193
	First radiation fractionation	DART-bid	16	4
		SABR	9	0
		Hypofracionated-RT	3	3
		Conventional-RT	3	1
	Re-rad fractionation	DART-bid	17	3
		SABR	9	1
		Hypofracionated-RT	2	3
		Conventional-RT	2	2
	Systemic therapy at first radiation (*n*)	yes	23	8
		no	8	0
	Systemic therapy at re-rad (*n*)	yes	16	2
		no	15	6
	Interval between radiation courses (months)	median	21	16
		range	9–80	6–145
	V20 _total lung_ (%) at re-rad	median	27	27
		range	3–43	3–53
	V25 _heart_ (%) at re-rad	median	4	1.5
		range	1–75	1–16.5

**Table 2:** Treatment related toxicity.

Toxicity (N = 39)						
Type of Toxicity		Grade 1	Grade 2	Grade 3	Grade 4	Grade 5
Acute	Esophagitis	n.a.	4	2	0	0
	Pneumonitis	n.a.	1	0	0	0
	Heart	n.a.	0	0	0	1
Late	Esophagitis	n.a.	0	0	0	0
	Pneumonitis	n.a.	0	0	0	0
	Hemorrhage	n.a.	0	1	0	0

n.a.: not assessed.

## Data Availability

The datasets used and/or analysed during the current study are available from the corresponding author on reasonable request.

## Supplementary Materials

The following are available online at https://www.mdpi.com/article/10.3390/curroncol28030170/s1, Table S1: Prognostic and predictive parameters related to overall survival, Table S2: Doses to organs at risk in both radiation courses except for V20_total lung_ and V25_heart_, which are given at the end of Table 1 “Treatment”.

## Abbreviations

CCI	Charlson co-morbidity index
CTCAE	Common toxicity criteria for adverse events
DART	Dose-differentiated accelerated radiotherapy
ECOG	Eastern cooperative oncology group
EQD2	Biologically equivalent dose in 2 Gy fractions
IMRT	Intensity modulated radiotherapy
LC	Local control
mOS	Median overall survival
MVA	Multivariate analysis
NSCLC	Non-small cell lung cancer
OAR	Organs at risk
OS	Overall survival
PS	Performance score
RT	Radiotherapy
SABR	Stereotactic ablative body therapy
SCLC	Small-cell lung cancer
UVA	Univariate analysis
VMAT	Volumetric intensity modulated arc therapy
