# Supplementary material for: Re-Irradiation for Locally Recurrent Lung Cancer: A Single Center Retrospective Analysis
Source: Curr Oncol. 2021 May 13;28(3):1835–46. doi: 10.3390/curroncol28030170 (PMC8161822; doi:10.3390/curroncol28030170)
Supplement: Supplementary file 1 [file curroncol-28-00170-s001.zip › curroncol-1179398-supplementary.pdf]

Current Oncology

MDPI

Article

Re-Irradiation for Locally Recurrent Lung Cancer: A Single Center Retrospective Analysis

Brane Grambozov, Evelyn Nussdorfer, Julia Kaiser, Sabine Gerum, Gerd Fastner, Markus Stana, Christoph Gaisberger, Romana Wass Michael Studnicka, Felix Sedlmayer and Franz Zehentmayr

Table S1. Prognostic and predictive parameters related to overall survival.

Uni- and Multivariate Analysis (Cox Regression)			
	**Variable**	**UVA**	**MVA**
Patient characteristics	Age	0.563	n.s.
	Sex	0.250	n.s.
	Weight loss	0.677	n.s.
	ECOG	0.137	n.s.
	Histology	0.205	n.s.
	T-stage	0.592	n.s.
	N-stage	0.125	n.s.
	M-stage	0.015	n.s.
	FEV1	0.526	n.s.
	COPD	0.733	n.s.
	Charlson Comorbidity Index	0.684	n.s.
Treatment related factors	Re-Irradiation volume	0.000	0.000
	Tumor location	0.070	n.s.
	Cumulative EQD_2	0.082	n.s.
	Interval between radiation courses	0.553	n.s.
	$V20_{total\ lung}$	0.427	n.s.
	$V25_{heart}$	0.936	n.s.
	Local control	0.005	n.s.
	Systemic treatment	0.497	n.s.

EQD_2: biologically equivalent dose in 2 Gy fractions; MVA: multivariate analysis; UVA: univariate analysis; $V20_{total\ lung}$: Total lung volume that receives 20 Gy or more; $V25_{heart}$: Heart volume that receives 25 Gy or more.

Table S2. Doses to organs at risk in both radiation courses except for $V20_{total\ lung}$ and $V25_{heart}$, which are given at the end of table 1 "Treatment".

Cumulative Dose to Organs at Risk		
V25 total lung (%)	median	21
	range	2–38
Mean lung dose (Gy)	median	15
	range	3–24
Mean esophageal dose (Gy)	median	24.6
	range	2–48
Dmax to central vessels (Gy)	median	70
	range	19–136
Dmax to spinal cord (Gy)	median	48
	range	12–69
